# Antimicrobial resistance preparedness in sub-Saharan African countries

**DOI:** 10.1186/s13756-020-00800-y

**Published:** 2020-08-28

**Authors:** Linzy Elton, Margaret J. Thomason, John Tembo, Thirumalaisamy P. Velavan, Srinivas Reddy Pallerla, Liã Bárbara Arruda, Francesco Vairo, Chiara Montaldo, Francine Ntoumi, Muzamil M. Abdel Hamid, Najmul Haider, Richard Kock, Giuseppe Ippolito, Alimuddin Zumla, Timothy D. McHugh, the PANDORA-ID-NET consortium

**Affiliations:** 1 University College London, London, UK; 2 University of Lusaka, Lusaka, Zambia; 3 University of Tübingen, Tübingen, Germany; 4 Duy Tan University, Da Nang, Vietnam; 5 National Institute for Infectious Diseases Lazzaro Spallanzani Institute for Hospitalization and Care Scientific, Rome, Italy; 6 Congolese Foundation for Medical Research, Brazzaville, Republic of Congo; 7 University of Khartoum, Khartoum, Sudan; 8 Royal Veterinary College, London, UK; 9 NIHR, UK

**Keywords:** Antimicrobial resistance, Joint external evaluation, One health

## Abstract

**Background:**

Antimicrobial resistance (AMR) is of growing concern globally and AMR status in sub-Saharan Africa (SSA) is undefined due to a lack of real-time data recording, surveillance and regulation. World Health Organization (WHO) Joint External Evaluation (JEE) reports are voluntary, collaborative processes to assess country capacities and preparedness to prevent, detect and rapidly respond to public health risks, including AMR. The data from SSA JEE reports were analysed to gain an overview of how SSA is working towards AMR preparedness and where strengths and weaknesses lie.

**Methods:**

SSA country JEE AMR preparedness scores were analysed. A cumulative mean of all the SSA country AMR preparedness scores was calculated and compared to the overall mean SSA JEE score. AMR preparedness indicators were analysed, and data were weighted by region.

**Findings:**

The mean SSA AMR preparedness score was 53% less than the overall mean SSA JEE score. East Africa had the highest percentage of countries reporting having AMR National Action Plans in place, as well as human and animal pathogen AMR surveillance programmes. Southern Africa reported the highest percentage of countries with training programmes and antimicrobial stewardship.

**Conclusions:**

The low mean AMR preparedness score compared to overall JEE score, along with the majority of countries lacking implemented National Action Plans, suggests that until now AMR has not been a priority for most SSA countries. By identifying regional and One Health strengths, AMR preparedness can be fortified across SSA with a multisectoral approach.

## Key points identified

- ‘Infection Prevention and Control’ (specifically in a clinical setting) was the strongest AMR category across SSA
- ‘Antimicrobial Stewardship’ was the weakest category across SSA
- Veterinary AMR surveillance and stewardship is less established than in clinical settings across SSA, so a multidisciplinary approach to improving these areas is needed to achieve One Health
- East Africa reported the strongest AMR response, thus lessons can be adapted from this region across the continent

## Introduction

The 68th session of the World Health Assembly in May 2015 [1] adopted the World Health Organization’s (WHO) global action plan on antimicrobial resistance (AMR), where AMR was included as a sustainable development goal to facilitate worldwide action to tackle a serious growing issue threatening global health [2]. Accurate data on AMR were unavailable worldwide and the expectations were that data collection, surveillance, and research on AMR would deliver quality data [3]. The five pillars of the WHO plan were to 1) improve awareness 2) obtain knowledge through surveillance 3) reduce the infection incidence 4) optimise antimicrobial use and 5) develop an economic case for sustainable investment needs for new medicines, diagnostic tools, vaccines, and other distinct interventions [1].

Western countries took up the challenges of the WHO action plan and several initiatives from Western Europe and the USA were established [4, 5]. From the data available, the rising trend in antibiotic-resistant bacteria appears to be reflected globally, with the increasing presence of methicillin-resistant *Staphylococcus aureus* (MRSA), extended-spectrum β-lactamase-producing (ESBL) *Enterobacteriaceae*, carbapenemase-producing *Enterobacteriaceae* (CRE), multi-resistant *Pseudomonas aeruginosa*, vancomycin-resistant enterococci, and multi-drug resistant *Acinetobacter baumannii* [6].

In contrast to high income countries, there are numerous additional challenges to implementing effective and sustainable AMR surveillance programmes in low and middle income countries such as those in Africa. These range from a lack of infrastructural and institutional capacities, lack of investment and human resources, underutilisation of available data and scarce dissemination to regulatory bodies [5, 7]. Routine AMR surveillance continues to be based on local hospital data, small cohort studies in neonatal and adult wards, routine laboratory samples taken from patients with suspected infection and health-care associated infections [8, 9]. Major data gaps remain on the issue of AMR in Africa including the actual burden of AMR in the community, hospital settings, animals and the environment, as well as microbial acquisition of AMR, transmission patterns, genotypic evolution of antimicrobial resistance mechanisms, clonal spread and asymptomatic carriage.

At a global level, the realisation of the increasingly serious nature of AMR has led to the formation of several initiatives to improve the surveillance and capture of AMR data. The WHO has created a number of AMR surveillance initiatives, including the tripartite database WHONET [10], the Advisory Group on Integrated Surveillance of Antimicrobial Resistance (AGISAR) and the Global Antimicrobial Resistance Surveillance System (GLASS) [11]. The WHO has identified a list of priority AMR pathogens to help address this, as shown in Table 1, taken from [12]. Amidst some controversy, tuberculosis was not included, despite growing antimicrobial resistance [5]. There is a need to strengthen the AMR evidence based data through proactive global surveillance and research and enhancing coordination and collaboration between African countries. This is the first step towards a true global action plan to tackle AMR with a multidisciplinary approach.

**Table 1:** List of WHO’s GLASS priority pathogens

WHO Priority Level	Species	Resistance pattern
Priority 1: Critical	*Acinetobacter baumannii*	Carbapenem-resistant
	*Pseudomonas aeruginosa*	Carbapenem-resistant
	Enterobacteriaceae*	Carbapenem-resistant, 3rd generation cephalosporin-resistant
Priority 2: High	*Enterococcus faecium*	Vancomycin-resistant
	*Staphylococcus aureus*	Methicillin-resistant, vancomycin intermediate and resistant
	*Helicobacter pylori*	Clarithromycin-resistant
	*Campylobacter*	Fluoroquinolone-resistant
	*Salmonella* spp*.*	Fluoroquinolone-resistant
	*Neisseria gonorrhoeae*	Third generation cephalosporin-resistant, fluoroquinolone-resistant
Priority 3: Medium	*Streptococcus pneumoniae*	Penicillin-non-susceptible
	*Haemophilus influenzae*	Ampicillin-resistant
	*Shigella* spp*.*	Fluoroquinolone-resistant

* Enterobacteriaceae include: Klebsiella pneumonia, *Escherichia coli*, Enterobacter spp., Serratia spp., Proteus spp., and Providencia spp., Morganella spp. Taken from [12]

The need for a One Health approach cannot be understated. Whilst the global threat of AMR has repeatedly been attributed to inappropriate use of antimicrobials in human and animal husbandry, AMR in animals and humans without previous exposure to antimicrobials has been observed. This highlights the complex evolution and transmission dynamics among people, domestic and wild animals and the environment [13–16]. Avoiding the horizontal transfer of AMR between these compartments is vital, as it is estimated that up to 75% of human infectious pathogens that have emerged or re-emerged are zoonotic [17]. The magnitude of environmental reservoirs, such as waste water, in which these pathogens might be harboured, plus the complications of climate change, urbanisation, anthropogenic activities, resource depletion and antimicrobial residues in the ecosystem further increases the danger of AMR transferral [18–20]. The importance of AMR surveillance is beginning to gain traction in Africa [9, 21, 22], although it is often difficult to identify whether data is collated, let alone what trends in prevalence exist. Implementing an effective antimicrobial stewardship programme poses a big challenge in real world settings and legislative knowledge is often low among physicians, pharmacists and veterinarians [13, 23].

**Fig. 1:**
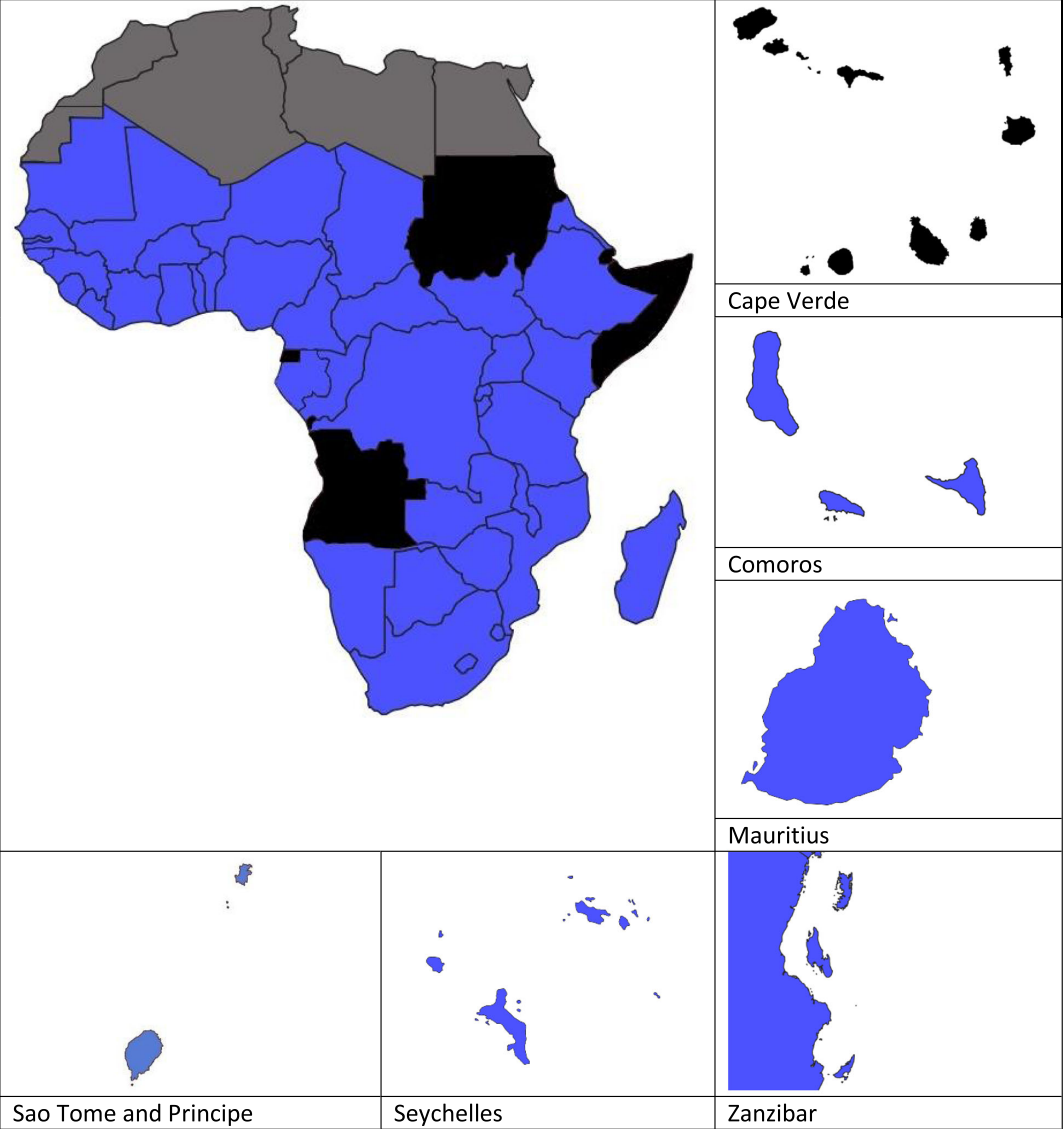
Maps showing the status of JEE completion of SSA countries. Black indicates that a country has not completed a JEE (at the time of writing Angola, Cape Verde, Djibouti, Equatorial Guinea, Somalia and Sudan are either in the process of completing or have not yet undertaken a JEE report), blue indicates a country that has a completed JEE. Countries in grey denote Northern African countries not included in this review

Alongside surveillance databases and committees, the need to identify country-specific preparedness for potential public health risks, including AMR, resulted in the publication of Joint External Evaluations (JEEs) [24]. JEEs are voluntary, collaborative processes to assess country capacities to prevent, detect and rapidly respond to public health risks. The target for AMR preparedness for countries is described as having ‘a functional system in place for the national response to combat AMR with a One Health approach’ [24]. Of the 50 SSA countries counted (as defined by WHO regions), as of March 2020, 44 had completed a JEE (86%). Figure 1 depicts the JEE completion status of the SSA countries.

JEE reports are broken down into four areas (‘prevent’, ‘detect’, ‘respond’ and ‘International Health Regulations (IHR) related hazards and points of entry’), and 19 sub-areas within these, one of which is ‘AMR’, which is further broken down into four categories. To help to identify whether a country has certain AMR indicators, a number of technical questions for each category are provided in the JEE for the country to answer, then scores are calculated based on the presence or absence of these indicators. A score of 1 denotes no capacity, 2 limited capacity, 3 developed capacity, 4 demonstrated capacity and 5 sustainable capacity.

Whilst tackling AMR can be broken down into many areas, as described in the JEEs, it is only by looking at the whole picture that effective gains can be made. JEE reports are created for individual countries and their results have thus far not been compared, to identify strengths and weaknesses across SSA. By exploring the JEE reports in more detail and comparing SSA countries and regions, it was possible to generate an overview of how the continent is working towards AMR preparedness. By breaking this down into African regions (West, Central, East and Southern), strengths can be pinpointed and adapted by countries who may need assistance. The knowledge gained from the JEE AMR preparedness score comparisons can be used to inform future AMR policies.

## Methods

**Fig. 2:**
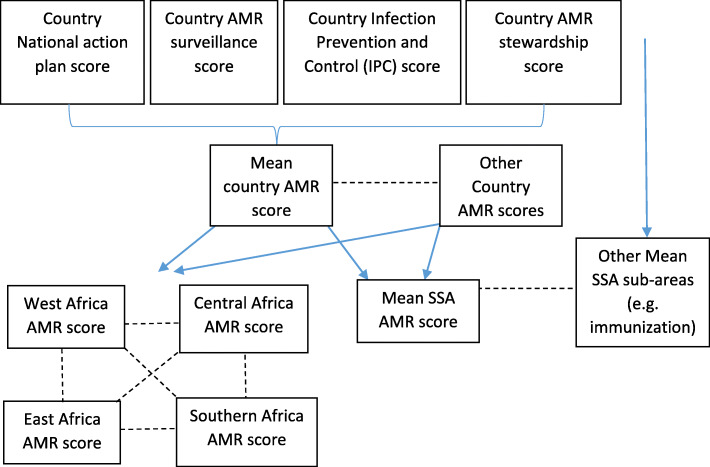
Explanation of how JEE data was analysed in this study. The mean of country AMR category scores was used as a ‘mean country AMR score’ (indicated by the blue solid line) and compared to other SSA countries (indicated by the black dotted line). The mean of all the ‘Mean country AMR scores’ was used as a ‘Mean SSA AMR score’ (indicated by the blue solid line) and then compared to other mean SSA sub-areas, e.g. immunisation (indicated by the black dotted line). ‘Mean country AMR scores were also weighted into regions (West, Central, East and Southern Africa) and compared (indicated by the black dotted line)

In this report, we aimed to identify the overall AMR preparedness across SSA using the scores from the JEE reports, so that strengths and weaknesses could be identified. To do this we analysed the 44 completed JEE reports from SSA countries, which were accessed between 6th November 2018 and 22nd March 2020 [25]. The mean SSA AMR score and SSA overall JEE score were calculated from all of the country mean AMR and JEE scores. To identify the performance of ‘AMR’ compared to other sub-areas across SSA countries, the mean SSA scores for the sub-area ‘AMR’ (the mean score for each category and each country) were compared to the mean SSA scores for each of the other sub-areas. An overview of how the data were analysed is outlined in Figure 2. One way ANOVA analysis was conducted to identify statistically significant differences (*p* < 0.05) between categories using GraphPad Prism 8.4.2.

The percentage of countries which fell into each score category was also calculated. Information from the accompanying technical questions was extracted and analysed to identify what percentage of the countries reported having AMR indicators in place. The other most commonly noted AMR structures were also collated and included, to give a deeper insight (Table 2). Although there were questions regarding animal Infection Prevention and Control (IPC), they were a new addition to the JEE technical questions, from the Second Edition [24] and as a result few countries provided a written response to them, so it was therefore not included as an indicator in this report. These indicator scores were weighted into African regions (15 West, 7 Central, 17 East and 5 Southern African countries, as defined by the United Nations) to highlight any particular patterns of AMR preparedness strengths. The guidelines for how scores are ascribed are outlined in the JEE Tool [24, 26].

**Table 2:** AMR preparedness categories and the indicators explored in this paper. Indicators are either taken directly from the scoring table (see the JEE tool [24, 26]) or they were from technical question answers from the technical questions

Category	Indicator	Source
Effective multisectoral coordination on AMR and the national action plan (in this paper referred to as ‘National Action Plan’)	Is there a National Action Plan in place?	Scoring table within the JEE tools document
AMR surveillance (in this paper referred to as ‘AMR surveillance)	Are human pathogen samples routinely tested for AMR?	Technical questions
	Are animal pathogen samples routinely tested for AMR?	Technical questions
	Is there a national human pathogen surveillance system in place?	Scoring table within the JEE tools document
	Is there a national animal pathogen surveillance system in place?	Scoring table within the JEE tools document
	Is there a national AMR reference laboratory?	Technical questions
Infection Prevention and Control (in this paper referred to as ‘IPC’)	Are there sufficient Water, Sanitation and Hygiene (WASH) programmes in place across all healthcare facilities in the country?	Scoring table within the JEE tools document
	Are there national training programmes (e.g. at higher education institutes on IPC?	Technical questions
Optimise use of antimicrobial medicines in human and animal health and agriculture (in this paper referred to as ‘Antimicrobial stewardship’)	Are there guidelines in place for the use of antimicrobials?	Scoring table within the JEE tools document
	Is there legislation in place for the distribution and use of clinical antimicrobials?	Technical questions
	Is there legislation in place for the distribution and use of veterinary antimicrobials?	Technical questions

## Results

**Fig. 3:**
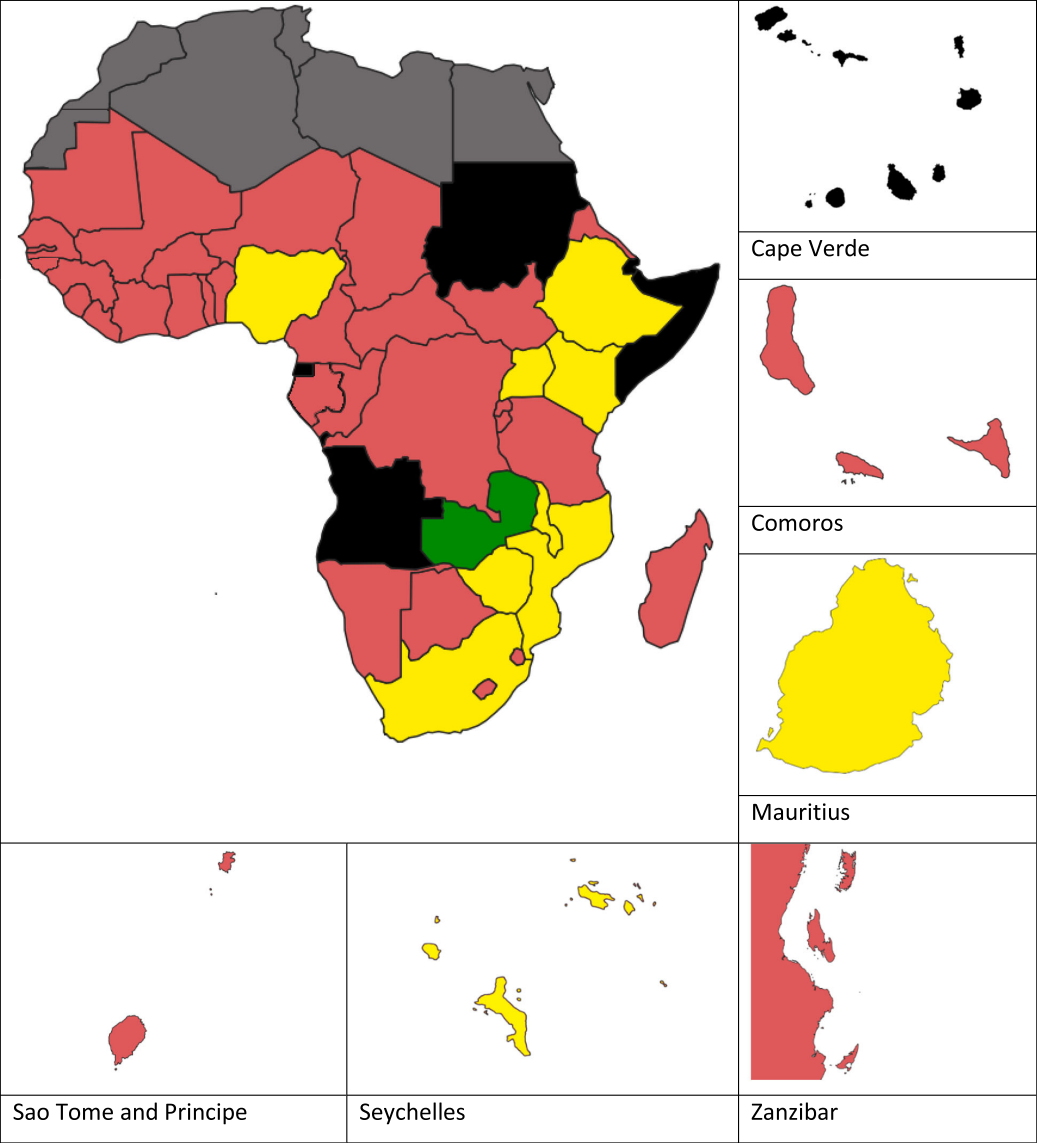
Map showing SSA country mean AMR JEE scores by colour category. Black denotes a country that has not completed a JEE and grey denotes North African countries not included in this review. Red indicates a JEE score of 1 (‘no capacity’). Yellow indicates a score of 2 or 3 (‘limited capacity’ or ‘developed capacity’) and green indicates a score of 4 or 5 (‘demonstrated capacity’ or ‘sustainable capacity’)

The mean SSA ‘AMR’ score was 1.42 (range 1.00–3.50), ranking it 17th among the 19 sub-areas when all sub-area mean scores for SSA were calculated (Table 3). This was 53% lower than the overall mean JEE SSA preparedness score of 3.05 (range 2.31–4.17). Figure 3 shows the mean AMR score for each country by colour category, as described in the JEE tool document. When compared, there was significance difference between the sub-areas (*p* = < 0.0001).

**Table 3:** Mean sub-Saharan African JEE scores when weighted by sub-area

JEE Area	JEE Sub-area	Mean SSA score	Ranking
Prevent	National legislation, policy and financing	1.45	15
	IHR coordination, communication and advocacy	1.91	10
	**Antimicrobial resistance**	**1.42**	**17**
	Zoonotic diseases	2.35	5
	Food safety	1.91	10
	Biosafety and security	1.63	12
	Immunization	3.38	1
Detect	National laboratory system	2.44	4
	Real time surveillance	2.90	2
	Reporting	2.26	7
	Workforce development	2.50	3
Respond	Emergency preparedness	1.42	18
	Emergency response operations	1.92	9
	Linking public health and security authorities	1.98	8
	Medical countermeasures and personnel deployment	1.33	19
	Risk communication	2.30	6
IHR other	Points of entry	1.43	16
	Chemical events	1.57	13
	Radiation emergencies	1.51	14

The mean of each countries’ scores for all questions in each sub-area was calculated, then the mean of all of the country means was calculated. The AMR sub-area is highlighted in bold. The rankings were calculated based on the mean score for each category for the 44 SSA countries.

When the mean of each AMR category was calculated there was a significant difference between the mean SSA category scores (*p* = 0.0207). ‘IPC’ had the highest mean score of 1.70 (range 1.00–5.00), while ‘Antimicrobial Stewardship’ had the lowest mean score of 1.23 (range 1.00–3.00).

Table 4 lists the mean SSA country score for each category and what percentage of countries scored 1–5.

**Table 4:** Percentage of countries who scored 1–5 for each category

Score	National Action Plan	AMR Surveillance	Infection Prevention and Control	Antimicrobial Stewardship
**1**	73%	77%	57%	82%
**2**	14%	16%	20%	14%
**3**	11%	5%	20%	5%
**4**	2%	2%	0%	0%
**5**	0%	0%	2%	0%
**Mean score**	1.43	1.32	1.70	1.23
***P*** **value**	*p* = 0.0207			

A score of 1 indicates no capacity, 2 indicates limited capacity, 3 indicates developed capacity, 4 indicates demonstrated capacity and 5 indicates sustainable capacity. The majority of countries scored 1 in each AMR category.

When countries were weighted by region, East Africa had the highest score when averaged across all AMR categories. Table 5 shows the mean regional scores for each AMR category, as well as whether there were significant differences between regions within each category. Only East Africa scored a category mean of > 2, in the ‘IPC’ category.

**Table 5:** SSA mean AMR category scores by region

African region and total countries within it		National Action Plan	AMR Surveillance	Infection Prevention and Control	Antimicrobial Stewardship	Regional AMR mean	Regional overall JEE mean
**West**	15	1.20	1.07	1.53	1.07	1.22	2.15
**Central**	7	1.29	1.00	1.14	1.00	1.11	2.00
**East**	17	1.71	1.65	2.18	1.47	1.75	2.38
**Southern**	5	1.40	1.40	1.40	1.20	1.35	2.40
***p*** **value**		*p* = 0.3168	*p* = 0.0407	*p* = 0.0493	*p* = 0.0866	*p* = 0.0207	*p* = 0.0113

Total countries within each region are those with JEE scores. Countries without JEE scores were not included in this table.

Each AMR category identified preparedness indicators, but responses to each of the technical questions were not uniform. National Action Plans for AMR are in place for 25% of SSA countries. 32% stated they conducted routine clinical pathogen AMR surveillance, as opposed to one country (2%) stating that they conducted routine veterinary pathogen AMR surveillance. Many countries reported that they conducted ad hoc, or research-based surveillance studies, but did not have a national programme in place. 66% of countries routinely collected and tested human pathogen samples for AMR and 25% collected and tested animal pathogens for AMR. The majority (59%) of SSA countries reported sending their AMR samples to a dedicated AMR National Reference Laboratory.

‘Infection Prevention and Control’ had the highest mean SSA category score and 25% of countries reported that they conduct training on AMR in an IPC capacity. Most countries (95%) did not have fully functional WASH or environmental health standards in place across all healthcare facilities. This category also had technical questions relating to animal IPC, although only six countries (Central African Republic, Chad, Ethiopia, Malawi, São Tomé and Príncipe and Zanzibar) specifically referred to animal IPC, and only Central African Republic mentioned animal IPC as a ‘strength’ rather than a ‘challenge’. The majority of answers from the technical questions centred around health care-associated infections.

For ‘Antimicrobial Stewardship’, 32% of countries stated that they had national guidelines for the appropriate distribution and use of antimicrobials. Prescription-only rules for the clinical use of antimicrobials were reported in 43% of countries, but the percentage of countries reporting legislation of antimicrobials for veterinary use was lower (32%). Most countries who reported having legislation in place stated that despite these regulations, issues with counterfeit drugs and the lack of enforcement was a problem. Some countries are in the process of making antimicrobials prescription-only, whilst others report extensive concerns over the uncontrolled use of unregulated and counterfeit antimicrobials. This is especially true for the veterinary sector, with antimicrobials often reported to be sold in village shops manned by store workers untrained in antimicrobial use. Antimicrobials were often reported to be used as a supplement to enhance growth and prevent diseases in poultry farms and beef and dairy production, although a few countries reported that antimicrobial use for animal growth promotion had been banned.

When assessing the AMR preparedness indicators by region, East Africa had the greatest number of countries with a National Action Plan (41%), national human and animal pathogen AMR surveillance programmes (65 and 47%, respectively) and routine animal pathogen AMR testing (47%) (Table 6). Southern Africa had the highest percentage of countries reporting routine human pathogen AMR testing, whilst Central Africa had the greatest number of countries with a national AMR reference laboratory (86%). Southern Africa scored highest of the regions for IPC training (60%), but East Africa was the only region with countries reporting to have functional WASH facilities in line with national standards (12%). Southern Africa also reported the highest percentage of countries with antimicrobial legislation for clinical and veterinary use in place and 40% of Southern African countries had antimicrobial usage guidelines.

**Table 6:** The percentage of countries, overall and broken down by region, who stated that they had AMR indicators present in their technical question answers

	SSA region, including number of countries				
	All SSA countries	West	Central	East	Southern
	(44 countries)	(15 countries)	(7 countries)	(17 countries)	(5 countries)
AMR indicators					
National Action Plan in place	11 (25%)	2 (13%)	1 (14%)	7 (41%)	1 (20%)
Human pathogen AMR surveillance	17 (39%)	3 (20%)	3 (43%)	9 (53%)	2 (40%)
Animal pathogen AMR surveillance	1 (2%)	0 (0%)	0 (0%)	1 (6%)	0 (0%)
Human pathogen AMR testing	29 (66%)	11 (73%)	3 (43%)	11 (65%)	4 (80%)
Animal pathogen AMR testing	11 (25%)	2 (13%)	0 (0%)	8 (47%)	1 (20%)
National AMR laboratory	27 (61%)	7 (47%)	6 (86%)	10 (59%)	4 (80%)
IPC prevention and control training	11 (25%)	3 (20%)	2 (29%)	3 (18%)	3 (60%)
Sufficient WASH programmes in place	2 (5%)	0 (0%)	0 (0%)	2 (12%)	0 (0%)
Drug stewardship framework	11 (25%)	3 (20%)	1 (14%)	5 (29%)	2 (40%)
Clinical antimicrobial legislation	19 (43%)	5 (33%)	2 (29%)	7 (41%)	5 (100%)
Veterinary antimicrobial legislation	14 (32%)	5 (33%)	2 (29%)	3 (18%)	4 (80%)

## Discussion

JEEs are powerful tools for identifying strengths and weaknesses in a country’s ability to deal with global health risks, as they present a defined set of indicators against which all countries can be compared. By comparing SSA country JEE scores and identifying where AMR sits in comparison to other JEE categories, we have shown that, whilst much work needs to be done to bring AMR in line with other areas, such as immunisation, there are countries and regions who have successfully implemented AMR control initiatives. The low mean SSA AMR preparedness score compared to the SSA JEE preparedness score suggests that until now AMR has not been a priority for most SSA countries, compared to the other sub-areas. The fact that the majority of countries lack an AMR National Action Plan suggests that they may have been lacking a focussed and coordinated response, although many stated that they are beginning to prepare and implement them, which is a positive step forward in the fight against AMR. With the lowest mean AMR category score, ‘Antimicrobial Stewardship’ needs the greatest JEE score improvement to align it with the other AMR categories. Focussing attention on antimicrobial stewardship will improve countries’ AMR preparedness scores and bring AMR in line with the other sub-areas, such as immunisation. Whilst it could be argued that AMR may not necessarily be a problem on the same scale as other public health issues, without national surveillance in place it is very difficult to tell the true extent of the problem.

The technical questions provide a deeper insight into the facilities in place, and yet to be achieved for each country. A constraint of this study was that the analysed indicators depended on the depth of the written response of each country, and whether the indicator was mentioned as being present or not. Whilst most countries mentioned whether they had, or were in the process of writing a National Action Plan for instance, fewer countries mentioned whether they conducted IPC training, and in general, veterinary indicators had fewer and less detailed responses. The lack of veterinary responses to these technical questions suggests that veterinary professionals might not yet be fully integrated into many countries’ public health response teams, however the creation of a multisectoral approach is a prominent part of the WHO’s Global action plan for AMR, which should address this [27].

There needs to be wilful political commitment to address AMR, including designated funding and the implementation of a fully multidisciplinary National Action Plan if countries are to make maximum use of their clinical and veterinary facilities. With the majority of countries reporting ‘no capacity’ for ‘AMR Surveillance’, capacity needs to be built nationally and regionally to obtain the necessary surveillance levels for key human and animal pathogen AMRs, for example those identified in the GLASS [28]. Many JEEs report that although countries don’t currently undertake national AMR testing, some do have significant laboratory capacities already in place, which could be quickly utilised in the future. This capacity needs to include susceptibility assays, training of diagnostic staff in testing methods and the implementation of quality control protocols [4, 5, 29]. To ensure this is sustainable and attainable, a stepwise approach should be used [30].

Some countries reported doing small-scale studies, thus some data on AMR are being collected, but this is often not translated into country-wide AMR surveillance with government-level reporting. The increasing deployment of app-based digital pathogen and case reporting, such as the surveillance and outbreak response management system (SORMAS) should make surveillance and reporting easier [31]. A systematic approach needs to be developed through routine data collection and enrolment of more surveillance sites for increased capacity. In most countries, there is a need to strengthen the One Health aspect of surveillance and incorporate veterinary and environmental monitoring into any existing clinical programmes, although recent publications suggest that this is now at least a consideration for some countries [21, 29]. Enrolling in the WHO GLASS can help countries identify priority needs.

Although this study has shown that ‘Infection Prevention and Control’ had the highest SSA average score out of the four AMR categories, it still has room for improvement. As part of the National Action Plan, a national IPC programme for human health, animal health and food production (including policies, guidelines and dissemination strategies) must be implemented, so that a One Health system for integrated assessments of the safety and functionality of facilities for public health emergencies is in place. Steps are being made, as AMR is mentioned in the IPC guidelines published by WHO AFRO [32]. Although the vast majority of countries reported having insufficient WASH or IPC programmes in their healthcare facilities, most did report having some level of IPC in most sites, especially in larger clinical sites. Functional IPC committees must be set up to cover all human and animal health facilities to ensure the training and awareness of health care professionals, whilst also ensuring that disinfectants, personnel protective equipment and suitable waste disposal systems are readily available so staff can carry out IPC successfully [33]. If good practice in the larger facilities can be reproduced in local, veterinary and environmental facilities, this should quickly boost the IPC programmes category score for SSA countries.

‘Antimicrobial Stewardship’ scored the lowest of all the AMR categories, suggesting that by focussing on this, real gains can be made in the fight against AMR. Whilst the introduction of antimicrobial stewardship has proved successful in some countries, and can be used as a template for others [34, 35], this study has shown that most countries still need to create and implement national guidelines on the appropriate distribution and use of antimicrobials in a One Health capacity to limit the risk of resistance transmission [14]. Increased national awareness of AMR and the legislated use of antimicrobials is required, and professional bodies should be instigated to regulate and educate the pharmaceutical practices of both human and animal healthcare professionals. This needs to be extended to antimicrobial retailers and field workers in communities, who are often at the forefront of antimicrobial dispensing. Updating a country’s essential drugs list and the laws regulating access to antimicrobials in clinical, veterinary and agricultural settings will help to ensure that legislation is correctly enforced. To aid this decision making, more research is needed to better inform treatment guidelines and importantly, to identify alternatives to antimicrobials as animal growth promoters.

With the two highest regional mean AMR scores, AMR preparedness lessons can be learned from both East and Southern Africa. East Africa, with the highest percentage of countries with multisectoral National Action Plans, collection of animal pathogen AMR data and both human and animal pathogen AMR surveillance, appears to have embraced the One Health approach and the importance of surveillance. Southern Africa has the highest percentage of countries with antimicrobial stewardship guidelines in place, as well as the highest percentage of countries with human and animal antimicrobial legislation in place.

## Conclusions

This study has compared the AMR section of the JEE reports for SSA to compare countries and regions and identify key strengths that can be adapted and utilised across the continent. The key points identified in this study suggest that SSA countries need to fully involve clinical, veterinary and environmental departments if they are to build a robust One Health AMR preparedness response. To do this, they must share their experiences and adapt the successful programmes from countries with strong AMR responses to suit their own unique needs to ensure a standardised and coordinated assault on AMR.

## Data Availability

The datasets analysed during the current study are available in the World Health Organization’s website repository. These datasets were derived from the following public domain resources: https://www.who.int/ihr/procedures/mission-reports/en/

## Abbreviations

AMR: Antimicrobial resistance
SSA: Sub Saharan Africa
JEE: Joint external evaluation
WHO: World Health Organisation
GLASS: Global Antimicrobial Resistance Surveillance System
IPC: Infection prevention and control
MRSA: Methicillin-resistant *Staphylococcus aureus*
ESBL: Extended-spectrum β-lactamase
CRE: Carbapenemase-producing *Enterobacteriaceae*
IHR: International health regulations
WASH: Water, sanitation and hygiene
